# Endoscopic treatment with lumen-apposing metal stent in a patient with esophageal stricture and esophagobronchial fistula: a case report

**DOI:** 10.3389/fmed.2025.1557738

**Published:** 2025-07-10

**Authors:** Mengmeng Zhang, Hao Tang, Yunlu Feng, Aiming Yang

**Affiliations:** Department of Gastroenterology, Peking Union Medical College Hospital, Chinese Academy of Medical Sciences and Peking Union Medical College, Beijing, China

**Keywords:** esophageal stricture, esophagobronchial fistula, lumen-apposing metal stent, endoscopic treatment, case report

## Abstract

Endoscopic interventions for postoperative esophageal strictures and fistulas are inherently challenging. The use of conventional self-expandable metallic stents has been restricted due to high migration rates. There has been no report about malignant esophageal stricture and postoperative fistula managed with a lumen-apposing metal stent (LAMS). We present a case of refractory postoperative anastomotic stricture and esophagobronchial fistula following esophagectomy. Endoscopic deployment of a LAMS effectively relieved the obstruction and sealed the fistula, with the patient tolerating the procedure well and experiencing sustained symptom resolution for 4 weeks. This case suggests the potential role of LAMS in maintaining luminal patency and sealing fistulas. Endoscopic ultrasound-guided LAMS placement should be a technically feasible, effective and minimally invasive approach for postoperative esophageal stenosis and fistula.

## Introduction

Esophageal stricture and fistula are serious postoperative complications following esophagectomy. Endoscopic interventions have relative advantages due to their minimal invasiveness, but still remain challenging, and no established guidelines exist for these methods.

Self-expandable metallic stents (SEMS) have been used widely to alleviate symptoms for malignant esophageal stricture and tracheoesophageal fistula (1, 2), proving more effective than simple drainage or nutritional support (3). However, the main drawback of SEMS is stent migration, ranging from 0 to 58%, which can lead to recurrent dysphagia and increasing risks of bleeding and perforation (1, 4). Another issue is the potential risk of tracheoesophageal fistula, a late complication that may occur post-SEMS placement, often necessitating additional overlapping covered metallic stents (1, 5).

The lumen-apposing, fully covered, self-expanding metal stent (LAMS) was initially introduced for endoscopic transluminal drainage. Its saddle-shaped design provides anchorage and imparts lumen apposition via its wide flanges and thus reducing migration risk (6). Here, we present a case of severe postoperative esophageal stenosis and esophagobronchial fistula successfully treated with endoscopic LAMS placement. The reporting of this study conforms to the CARE guidelines, and patient consent for publication was obtained.

## Case report

A mid-70s man with a history of adenocarcinoma of the gastroesophageal junction underwent esophagectomy with esophagogastro-anastomosis, complicated by a postoperative anastomotic leak. This complication gradually improved with jejunal feeding and thoracic drainage. He had no previous history of other chronic diseases or surgeries. Approximately 10 months post-surgery, the patient presented with severe dysphagia and malnutrition. Upper gastrointestinal imaging and endoscopy revealed an anastomotic stricture and esophagobronchial fistula. Multiple rounds of endoscopic dilations were performed, but symptom relieved lasted less than a month. We attempted endoscopic ultrasound (EUS)-guided LAMS deployment to resolve this problem.

**Figure 1:**
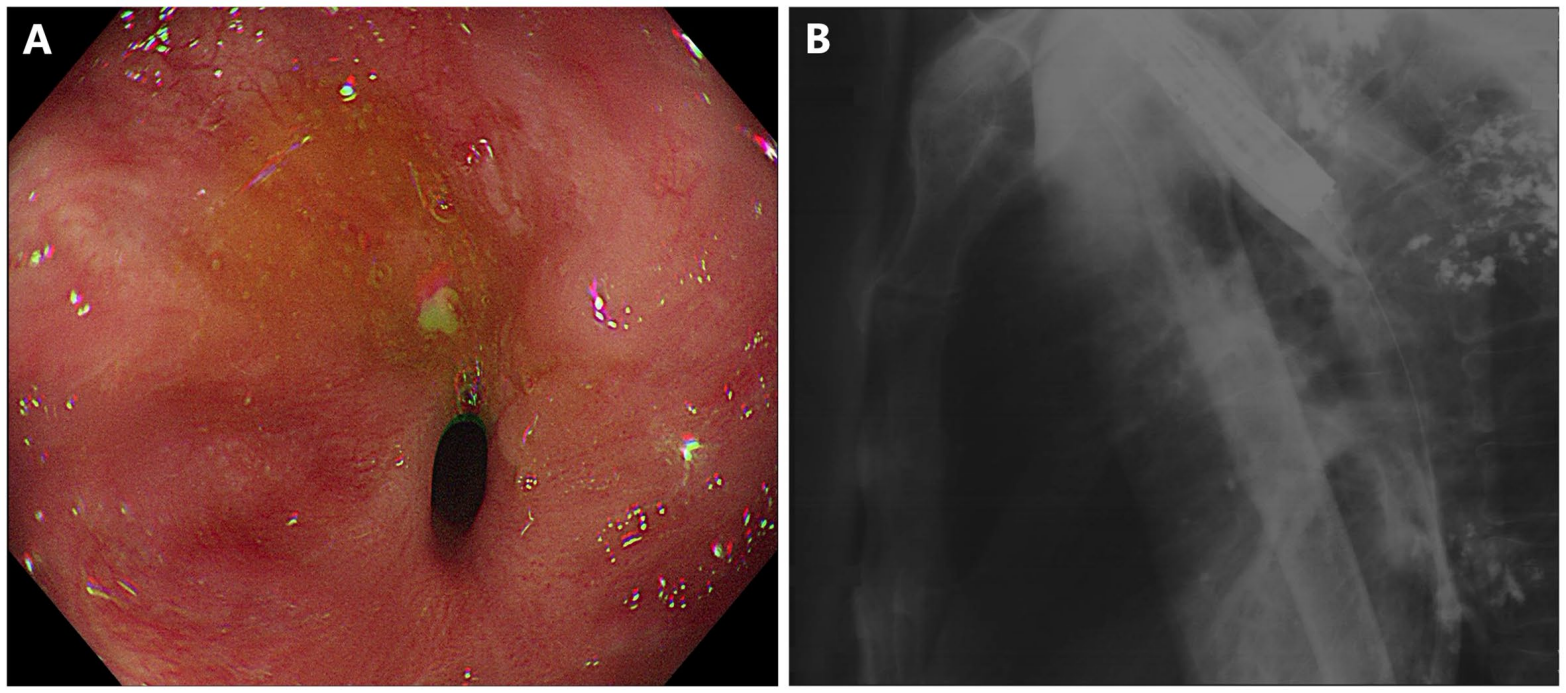
Short-segment anastomotic stricture and fistula in a patient with esophageal carcinoma after esophagectomy. **(A)** Esophagogastro-anastomosis stricture with an inner diameter of 3 mm. **(B)** X-ray radiography showed anastomotic leak connected to the right bronchus.

**Figure 2:**
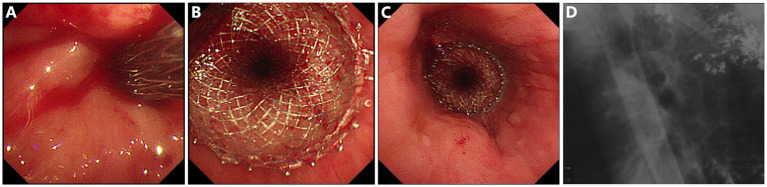
Placement of a lumen-apposing metal stent (LAMS) for esophagogastro-anastomosis stricture and fistula. **(A)** Insertion of the LAMS over the guide-wire. **(B,C)** Endoscopic view of the successfully placed LAMS. **(D)** LAMS *in situ* under X-ray radiography.

As shown in Figure 1, gastroscopy identified a stricture measuring 10 mm in length, with an inner diameter of 3 mm, at 25 cm from the incisors. X-ray radiography confirmed the anastomotic leak was connected to the right bronchus. A 15-mm LAMS (Hot AXIOS, Boston Scientific Corp., Marlborough, MA, United States) was deployed across the esophageal stricture (Figure 2). Briefly, a forward-viewing echoendoscope (TGF-UC260J; Olympus Medical Systems, Tokyo, Japan) was utilized for LAMS placement. Initially, a guidewire was advanced across the stricture through the accessory channel, followed by the introduction of the LAMS 10.8Fr catheter over the guidewire. Stent deployment was achieved through controlled, independent stepwise release of each flange under endoscopic visualization with fluoroscopic guidance. Immediately after LAMS placement, the patient’s dysphagia was relieved, and he was able to tolerate soft foods. There were no adverse events including bleeding or stent displacement.

Pathology of anastomotic biopsies showed squamous epithelium with mild to moderate dysplasia. 18F-FDG positron emission tomography/computed tomography (PET/CT) revealed multiple nodules with abnormally high FDG uptake near the anastomosis and splenic hilum. Due to tumor progression, the patient is currently receiving chemotherapy combined with immunotherapy in the oncology department. His digestive symptoms remain stable, and X-ray radiography indicates that the LAMS remains *in situ*.

## Discussion

This case demonstrates the successful LAMS placement for refractory malignant esophageal strictures and fistula, highlighting the advantages of LAMS in fistula sealing and low stent migration risk compared to other stents.

For esophageal stricture and fistula, SEMSs are primarily indicated to alleviate obstructive symptoms (7, 8). SEMS, typically measuring 4–12 cm in length and 6–10 mm in diameter, is composed of various metal alloys, sometimes added coverings. SEMS placement is a technically feasible and effective method for treating postoperative esophageal strictures (2, 9, 10). However, adverse events such as stent migration or esophageal injury are common. Reijm Agnes et al. (9) reported 46.2% of patients with malignant esophageal strictures treated with SEMS experienced at least one SEMS-related complication and 11.4% presented with stent migration. Similarly, SEMS migration rates for anastomotic leakage range from 19 to 53% (10, 11). Moreover, patients with refractory benign esophageal strictures treated with SEMS did not have a significantly longer dysphagia-free period or better outcomes compared to those treated with dilation (12).

LAMSs have been increasingly utilized for endoscopic treatment. Unlike the tubular SEMS, LAMS has a dumbbell-like structure, short saddle length, and large inner luminal diameter, which allows for lumen apposition, even pressure distribution, and reduces stent migration risk. The detailed technical parameters are illustrated in Supplementary Table 1. A multicenter study demonstrated that LAMS placement is a well-tolerated therapeutic option for luminal gastrointestinal stricture, with high technical success of 96.7% and a low stent migration rate of 8.0% (6). There are also some case reports of LAMS being used for benign esophageal strictures or fistulas. Barajas Pérez et al. (13) reported a case of benign esophageal stricture with iatrogenic perforation, where LAMS was successfully placed for 3 weeks after SEMS migration within 1 week, with the patient remaining asymptomatic post-LAMS removal. Most reported cases of LAMS used in esophageal strictures involve benign and short-segment strictures (14, 15).

To our knowledge, this is the first report of successful LAMS placement for malignant esophageal stricture concomitant with esophagobronchial fistula. Our findings may provide promising alterative procedure for postoperative esophageal complications. Firstly, the LAMS is advantageous in its low migration rate, potentially increasing clinical improvement and reducing the need for repeated stent exchanges. It was reported that approximately 90% of patients achieved symptomatic resolution with LAMS in place for a median of 60 days, and 82.6% could sustain without further intervention during a median follow-up of 100 days after stent removal (6). In addition, the potential adverse outcomes of LAMS are partially controllable. Postprocedural bleeding caused by local mucosal injury (16) and aortoesophageal fistula caused by localized ischemia or necrosis (17) are the underlying complications after LAMS placement. Therefore, it is recommended that LAMS size should be chosen based on the characteristics of the esophageal strictures, with caution advised when using the 20-mm LAMS, which has the largest flange diameter among all esophageal stents (17). The safety of LAMS in esophageal strictures and fistulas requires further investigation and more clinical experience.

## Conclusion

This case report demonstrates that LAMS placement is a technically feasible and effective endoscopic procedure for malignant postoperative esophageal strictures and esophagobronchial fistula, with advantages in reducing stent migration risk compared to conventional SEMS. Further data are needed to confirm the efficacy and safety of LAMS in the management of esophageal stricture and fistula.

## Data Availability

The original contributions presented in the study are included in the article/Supplementary material, further inquiries can be directed to the corresponding author.
