# Supplementary material for: Endoscopic treatment with lumen-apposing metal stent in a patient with esophageal stricture and esophagobronchial fistula: a case report
Source: Front Med (Lausanne). 2025 Jul 10;12:1557738. doi: 10.3389/fmed.2025.1557738 (PMC12286958; doi:10.3389/fmed.2025.1557738)
Supplement: Supplementary file 1 [file Table_1.pdf]

Supplementary Table 1 The technical parameters of self-expandable metallic stent (SEMS) and lumen-apposing metal stent (LAMS).

Indicators	SEMS	LAMS
Material	Metal alloy (e.g. Nitinol)	Metal alloy (e.g. Nitinol)
Morphology	Cylindrical with/without flared ends	Dumbbell-shaped
Design	Uncovered or covered	Fully covered
Lumen diameter	6, 8, 10 mm commonly	10, 15, 20 mm commonly
Length	60, 80, 100 mm commonly	10, 15 mm commonly
Expansion	Self-expanding	Self-expanding with radial force
Deployment Guidance	Fluoroscopy/endoscopy	Endoscopic ultrasound
